# Evaluation of an interactive program for preventing adverse drug events in primary care: study protocol of the InPAct cluster randomised stepped wedge trial

**DOI:** 10.1186/1748-5908-8-69

**Published:** 2013-06-19

**Authors:** Maud Keriel-Gascou, Karine Buchet-Poyau, Antoine Duclos, Muriel Rabilloud, Sophie Figon, Jean-Pierre Dubois, Jean Brami, Thierry Vial, Cyrille Colin

**Affiliations:** 1 Université de Lyon, Lyon F-69000, France; 2 Université Lyon 1, Département de Médecine Générale, Lyon F-69373, France; 3 Université de Lyon, EA Santé-Individu-Société 4128, Lyon F-69002, France; 4 Université Lyon 1, Medical School Lyon Est, Lyon F-69373, France; 5 Hospices Civils de Lyon, Pôle Information Médicale Evaluation Recherche, Lyon F-69003, France; 6 Hospices Civils de Lyon, Service de Bio statistique, Lyon F-69003, France; 7 CNRS, UMR 5558, Laboratoire de Biométrie et Biologie Evolutive, Equipe Biostatistique-Santé, Villeurbanne F-69622, France; 8 Université Paris Descartes, Faculté de médecine, Paris F-75006, France; 9 Hospices Civils de Lyon, Service de Pharmacovigilance, Lyon F-69003, France

**Keywords:** Patient safety intervention, Primary health care, Antihypertensive drug, Adverse drug event, Cluster randomised controlled trial, Stepped wedge

## Abstract

**Background:**

Adverse drug events could often be prevented. One of their main causes is that patients rarely know how to detect them. Another cause is inadequate communication between patients and physicians. If patients were to be effectively trained in detecting and reporting adverse drug events, this should help to prevent their occurrence and subsequent complications. Our purpose is to present the protocol of the InPAct trial, which aims to evaluate an interactive program that encourages patients to report adverse drug events in primary care.

**Methods/design:**

We will conduct a cluster randomised controlled stepped wedge trial, with eight clusters of 10 general practitioners each. The physicians will suggest to all of their antihypertensive-treated patients that they take part in this study. The InPAct program will be implemented in the clusters in random order along five successive three-month periods. Two new clusters will be trained in implementing the program at each step. The program features: an interactive patient booklet including informative paragraphs, several care plans and adverse drug event report forms; and standardised training of physicians in how to present the booklet to the patient. The primary outcome will be the reporting of adverse drug events by patients to their physician within three months. We assume that the number of patients reporting at least one adverse drug event will increase from 3% before program implementation to 7.5% afterward (coefficient of variation = 0.5, α = 0.05, β = 0.2), which means that 1,200 patients must be included. The effect of the intervention on the main outcome will be quantified and tested using a mixed logistic model to integrate cluster and time effects.

**Discussion:**

Our choice of a stepped wedge design is particularly appropriate for evaluating the implementation of a patient safety program within the constraints of general practice. We describe the InPAct intervention, which is an original program that is intended to improve communication between patients and physicians. Indeed, none of the previously published intervention studies has combined a patient education program and a patient reporting system for adverse drug events with the aim of improving patient safety in primary care.

**Trial registration:**

This study is registered in ClinicalTrials.gov NCT01610817.

## Background

According to a recent report of the Canadian Patient Safety Institute, the management of adverse drug events (ADEs) in primary medical care is a public health priority [1]. The proportion of patients presenting an ADE within the first three months following an ambulatory drug prescription was estimated to reach 25% in Gandhi et al. [2]. Cardiovascular drugs have been found responsible for 20% to 30% of hospitalisations for ADEs; meanwhile, 20% to 40% of ADEs could be prevented [2,3]. A lack of information and communication between patients and health care professionals has been identified as an underlying factor favouring the occurrence of ADEs [4,5]. A significant proportion of ADEs and associated complications could be prevented if risk situations were detected earlier and if patients were better informed about participating in their own medical care and about reporting ADEs [6]. In the InPAct (Information for Participating Actively) study, we focused on the reporting of ADEs by patients. Because clear differences have been shown between reports emanating from patients and those emanating from health professionals, it is essential that patients be able to report ADEs in order to supplement the reporting of ADEs by health professionals [7,8]. It has been found that patients report ADEs four times less often than do health professionals [7,9]. Considerable variations have been observed from one country to another in terms of the proportion of total ADE reports submitted by patients as opposed to the proportion submitted by health professionals [10]. In France, according to data from the ANSM (French National Agency for the Safety of Medicines and Health Products), only 1% of official ADE reports are made by a patient. We decided to focus on ADE reports made by antihypertensive-treated patients in primary care for three reasons. First of all, hypertension is common, with two-thirds of people older than 65 affected by this condition in developed countries [11]. Also, drug prescriptions for this sector of the population are often composed of at least four different drugs, which increases the risk of ADEs [12-14]. Finally, the proportion of patients experiencing at least one ADE within the first three months following the prescription of an antihypertensive has been estimated at 12.6% [2].

The InPAct intervention is an interactive program. It consists of educating patients about taking a more active role in their own care, so that they are more likely to report antihypertensive-related ADEs to their general practitioner (GP). Another important goal of the InPAct program is to improve patient safety in primary care.

Our purpose is to present the methodology and expected results of the InPAct trial. The primary outcome aims to evaluate the effectiveness of the InPAct program in promoting patient reports of ADEs. The secondary outcomes include the measurement of the InPAct program’s effectiveness in limiting ADE risk situations and in reducing the occurrence and severity of ADEs. The other secondary outcomes are the evaluation of the health knowledge and the level of satisfaction of antihypertensive patients with respect to the medical care provided by their GP.

## Methods/design

### Study design

The design of the InPAct protocol is a cluster randomised controlled (CRCT) stepped wedge trial design, which is appropriate for assessing the impact of patient safety interventions [15]. This design involves the sequential rollout of an intervention in the clusters over several time periods. The order in which the clusters will receive the intervention is determined at random, and by the end of the study, all clusters will have received the intervention. Finally, each cluster will successively be situated in a control group and then in an intervention group.

**Figure 1:**
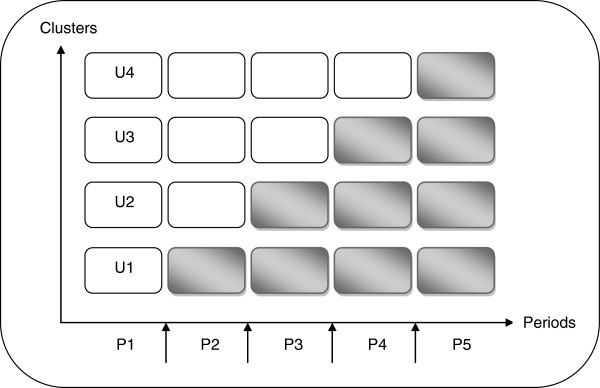
**InPAct study design.** One unit of randomisation (U1, U2…) contains two clusters of general practitioners, who implement the InPAct program during the same period of time (P2 to P5). Non-gray cells represent control units. Gray cells represent units in which the InPAct program was implemented.

In the InPAct protocol, eight clusters of several GPs were defined. Four steps, one unit (U) of two clusters per step, and five periods (from P1 to P5) of three-month duration were defined. In the first period, the InPAct program will not be implemented. The InPAct program will be rolled out sequentially to the four GP cluster units over the last four time periods (Figure 1). The order in which the clusters will take part in the program is determined at random, allowing the full implementation of the intervention by all participating GPs by the end of the trial [16,17].

### Setting

The InPAct program will take place in general practitioners’ offices of several suburban cities in three French regions (Rhône–Alpes, Auvergne and Ile-de-France).

### Recruitment of general practitioners

Eight clusters were defined, with ten GP volunteers per cluster. The GPs were grouped in a cluster according to criteria of geographic proximity, practice in the same medical centre, or interaction among professionals.

### Recruitment of patient participants

GP investigators involved in the study are required to recommend participation in this study to each patient over 18 years old who is consulting to initiate, modify or renew an antihypertensive drug prescription. In order to be included, the patients must meet the inclusion criteria, understand the investigator’s language, and agree to answer a telephone survey at home. Only the patients included in the study after the entry of their GP into the intervention group will receive the interactive booklet. Each patient will be followed during three months, with the outcome criteria collected at the end of this period.

### InPAct program

The InPAct program consists of providing an interactive booklet to the patients as well as standardised training to the GPs regarding the presentation of the booklet to the patient. A pilot study involving a small sample of patients and GPs has already made it possible to test the feasibility and acceptability of InPAct program implementation. This pilot study has provided some arguments in favour of a beneficial effect of the intervention [18].

### 1. InPAct booklet description

Ten steps were necessary to develop the InPAct interactive booklet [18]. This work was performed by a collaborative group including patients, GPs and other health professionals involved in ambulatory care or who specialised in one of the medical domains of the study. The initial booklet was created using the Delphi consensus method [19]. In a following step, a qualitative study made it possible to improve booklet content and booklet presentation instructions for GPs. Several tests of text readability and booklet reviews by communications professionals were repeated during the booklet creation process. The booklet was printed on both sides of a thick, film-coated, glossy A4-size paper folded into thirds. The title and instructions for use are on the outside. Informative paragraphs on hypertension, cardiovascular risk factors, situations involving a risk of ADEs and a list of the most frequent antihypertensive-associated ADEs are inside. Several ADE report forms and several care plans that can be annotated by the patient are included in the form of memo pads placed within the booklet.

### 2. General practitioner training

GP investigators will receive booklets and will participate in a web seminar explaining the kind of oral information to provide when the booklet is given to the patient. As determined by the pilot study, physicians are encouraged to give the booklet to the patient and to first present the principal value of the booklet for the patient, that is, the reporting of ADEs for increased patient safety [18]. In order to increase physician-patient interaction and communication, physicians are encouraged to first ask several questions to evaluate patient knowledge of the benefits and risks of the antihypertensive drug prescription, and to then give some complementary information. GPs are asked to present the care plan to the patient and to explain the antihypertensive drug prescription. Also, GPs are incited to explain the lab tests planned for the patient follow-up and to present to the patient the open text box for patient questions in the care plan. Once the booklet has been presented to the patient in this manner, GPs must again remind the patient of the main value of the booklet and describe the content of the ADE report form. Our hypothesis is that the InPAct booklet should facilitate the interaction between GPs, who are primary healthcare professionals, and their patients, leading to improve ADE reporting by patients.

### Comparator

**Figure 2:**
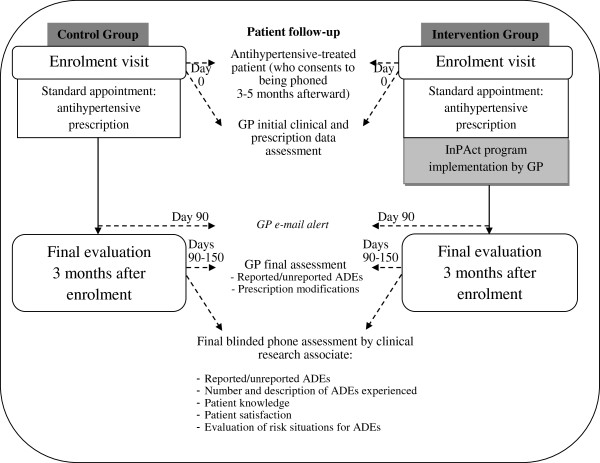
Diagram of the management of patients included in the InPAct study in both groups.

The care management of antihypertensive-treated patients carried out by GPs belonging to the control group (*i.e.*, prior to InPAct program implementation in their cluster) will be identical to the usual practice. All outcomes will be assessed three months after patient enrolment, partly by the GP and partly through a phone survey of the patient. All outcomes will be identically collected in both control and intervention groups (Figure 2).

### Outcome measurement

The primary outcome of the InPAct study is the reporting of ADEs by antihypertensive-treated patients to their GP during the three-month period following enrolment. Patient reporting was defined as all patient reports to the GP of a medical issue for which the patient suggested a drug to be the cause. The secondary outcomes are: ADE-associated medical complications presented by antihypertensive-treated patients (*e.g.*, orthostatic hypotension, falls, renal failure, hydroelectrolytic disorders, dehydration, confusion syndrome, heart rhythm disorder, or hospitalisation); ADEs presented by antihypertensive-treated patients (reported or not); situations presenting a risk of ADEs for antihypertensive-treated patients; modification of the prescription by the GP, following an ADE; a patient knowledge evaluation (on hypertension and corresponding care, on benefits, risks and management of risk associated with drug prescriptions); and an evaluation of patient satisfaction.

### Data collection

When a patient is recruited for the study, the GP will need to enter the patient’s clinical data and prescription into an electronic case report form (e-CRF) (Figure 2). After three months of patient follow-up, the GP will have to collect data on ADE occurrence, patient ADE reporting and prescription modifications, for entry into the e-CRF. Also after three months of patient follow-up, clinical research associates are to interview patients using a standardised phone questionnaire dealing with ADE occurrence, situations presenting a risk of ADEs, ADEs that occurred without being detected by GPs, and patient skills and satisfaction concerning their medical care. The associates will have to enter the corresponding data into the e-CRF and will have to provide a detailed explanation of all drug prescriptions made by the GP.

### Sample size

Sample size calculations were carried out using the method implemented by Hussey and Hughes [20]. In our study, the number of clusters was fixed at eight, and the number of time periods was fixed at five. The expected effect of the intervention is an absolute increase of 4.5% of the percentage of hypertensive patients who will report at least one ADE; *i.e.*, an increase from 3% in the group without intervention to 7.5% in the group with intervention. The clustering induces an increase in the variance of the outcome criterion, due to inter-cluster heterogeneity; this increase is quantified by the inter-cluster coefficient of variation. For a given number of patients per cluster, the power of the study was calculated for different coefficients of variation between 0.1 and 0.5, which corresponds to the range of values usually observed. The inclusion of 240 patients per time period (30 patients per cluster) leads to a power greater than 80% regardless of the coefficient of variation (Table 1). The type I error was fixed at 5% for a bilateral test. In the context of the InPAct study, for logistical reasons and to avoid the risks of inter-cluster contamination and clusters with no inclusion, it was preferable to limit the number of GP clusters. With a classic CRCT parallel group design, the number of patients to include would have to be at least 6,000 to maintain a statistical power of 80% with 12 clusters (under 12 clusters it is impossible to reach a statistical power of 80%, regardless of the number of patients), but with our choice of stepped wedge design, the patient number decreases to 1,200 for eight clusters with a statistical power of 80%.

**Table 1:** Statistical power calculated from the coefficient of variation and number of patients per time period

**Cv**	**184 patients per time period**	**208 patients per time period**	**240 patients per time period**
0	0.80	0.85	0.89
0.1	0.80	0.84	0.89
0.2	0.78	0.83	0.87
0.3	0.76	**0**.**81**	0.85
0.4	0.74	0.78	0.83
0.5	0.72	0.76	**0**.**81**

The expected percentage of hypertensive patients reporting at least one ADE is determined as follows: the proportion of patients with ADEs within the first three months of antihypertensive treatment has been estimated at 12.6% in Gandhi et al*.*[2]; and the frequency of ADEs reported by patients has been estimated to be four times lower than that reported by health professionals and seven times lower than that reported by GPs [7,9]. Our hypothesis is that when four patients experience an ADE, only one reports it. We have therefore estimated that prior to the implementation of the InPAct program, 3% of antihypertensive-treated patients would report an ADE.

### Randomisation

Before the beginning of the implementation phase, clusters were randomly allocated a time when they are to be given the intervention. In agreement with the CONSORT statement extended to cluster randomised trials, the team of statisticians, which was blinded to the identity of the clusters, randomised the clusters using computer generated random numbers [21]. The randomisation sequence was therefore established before the beginning of program implementation. Only the research team was aware of the allocation of all of the clusters. In order to maintain allocation concealment, GPs will be informed of their allocation time only in the month prior to the date of implementation in their cluster. This one-month delay is necessary for the organisation of their training for the InPAct program.

It is impossible to blind patients and GP participants for the intervention, due to the nature of the intervention, *i.e.*, training GPs in the distribution of a booklet to patients. Outcome assessment will, however, be essentially blinded. Indeed, most of the data are collected in a blinded fashion by clinical research associates, whereas the only non-blinded medical data will be those collected by the GPs.

### Statistical analysis

The quantitative characteristics of the patients included during the control and intervention periods will be described either by using the mean and the standard deviation, or by using the quartiles and the minimum and maximum values, depending on the shape of the distribution. The qualitative characteristics of the patients will be described using the absolute and relative frequencies in the different categories. The comparisons between the two groups will be carried out using a student-*t* test or a non-parametric test for the quantitative characteristics and a chi-squared test or a Fisher’s exact test for the qualitative characteristics.

A stepped wedge design is a unidirectional crossover trial. The particularity of this design, in comparison with traditional crossover studies, is that the information allowing quantification and testing of the intervention effect is present within and between the clusters. As recommended by Hussey and Hughes [20], the analysis of the intervention effect on the main outcome will be carried out using a mixed logistic model with a random intercept, in order to take into account the within-cluster correlation, to use both within-cluster and between-cluster information, and to take into account any evolution of the intervention effect over time. The mixed logistic model may be used to analyse a cluster randomised trial even when the number of clusters is small, as long as the number of patients per cluster is greater than approximately 30, which is the case in our study. Austin showed that the estimation of the regression coefficient, which quantifies the intervention effect, preserves good properties in terms of bias and coverage probability of the confidence interval, if the number of subjects per cluster is over 30, and even if the number of clusters is as few as five [22]. This analysis will be carried out using the lme4 package of the most recent version of the R software.

### Ethical consideration

GP investigators must recommend that all patients who are consulting for antihypertensive prescription consider participating in this study, without conducting any selection of patients to be approached or not. The participating patients will give their written informed consent after being told about the study. Their participation or refusal will not affect their medical and nursing care.

### Trial status

The InPAct study is funded by the French National Program for Hospital Clinical Research. The study protocol was reviewed and approved on June 28, 2011, by the Hospital Ethics Committee. All procedures are in accordance with the Declaration of Helsinki. The InPAct study has been registered as a clinical trial (Current Controlled Trials NCT01610817). The enrolment of patients has begun, but data cleaning or analysis had not yet begun as of this article’s submission.

## Discussion

This article has outlined the design and method of data collection for the InPAct study, as well as details on the implementation of the intervention. The aim of this study is to evaluate how effectively the InPAct program encourages patient-GP interaction and improves patient-initiated ADE reporting. This is the first randomised controlled study that combines a patient education program and a patient reporting system for ADEs with the aim of improving patient safety in primary care.

### Originality of the InPAct program

The InPAct program is a patient safety and patient education intervention that has been developed and evaluated through successive qualitative and quantitative studies as recommended by the Medical Research Council [23]. The implementation of the InPAct program may increase ADE reporting by patients in general practice. In order to improve communication, the InPAct program will encourage GPs to ask their patients about their condition, their antihypertensive prescription drugs, their other healthcare prescriptions, and their experience with ADEs. The GP will inform the patient about the benefits and risks of antihypertensive prescription drugs. The patient will be encouraged to participate actively in his care by writing a care plan and in his own safety by detecting and describing ADEs using the report form included in the InPAct booklet. The GP will analyse patient reports to establish what role, if any, a particular drug may have played in the onset of an ADE. It is expected that the detection of ADEs and their description in report forms by patients will promote the implementation of corrective and preventive actions to reduce the occurrence of ADEs and their complications in general practice. Interventions to improve drug management in general practice have already been tested. Weingart et al. have shown in an observational study that the reception of an automatic email with information about ADE interactions and allergies within the first 10 days of a new prescription can improve communication between a patient and his GP [24]. Roughead et al. have demonstrated that educational materials for GPs and patients can improve the verification of the prescription by patients at home [25]. Fick et al. have shown that an educational booklet with a list of inappropriate medications for the elderly can improve a review of prescriptions by GPs [26]. In these general practice intervention studies, however, patients were not encouraged to participate actively in their care, and professional practice was assessed instead of patient outcomes.

A program combining both oral and written information can foster improved communication between a patient and his GP. Such a program can also provide patients with enhanced skills for participating in their own care. It has been shown that information leaflets can improve antibiotic use for respiratory tract infection care management in general practice [27], and that self-management leaflets and booklets for minor illness can reduce the number of inappropriate consultations [28]. Moreover, educational leaflets and questions on knowledge about contraception [29] or hypertension [30] can improve not only patient knowledge, but also patient compliance. Finally, we have identified only one study that has demonstrated the effectiveness of patient education in reducing the frequency of ADE occurrence [31]. We have not identified other educational patient programs that have shown a statistically significant improvement in ADE prevention. None of these studies featured a combination of a patient education program and a patient reporting system for ADEs that aimed to prevent ADEs and reduce their associated complications.

### Advantages of the stepped wedge design in the particular context of a study in general practice

The stepped wedge trial design used in this study is a particular form of cluster randomised controlled design that is especially useful for the evaluation of patient safety programs in general practice. Findings from such a study design have been shown to generate sound scientific evidence and may make it easier to implement complex health interventions. The CRCT stepped wedge design can prove the effectiveness of an intervention with more robustness than can non-randomised designs, in particular in the case of routine implementation. This design is particularly relevant when for logistical, practical or financial reasons, it is difficult to deliver the intervention simultaneously to all of the clusters [15-17].

First, in the InPAct study, it was inappropriate to use individual randomisation of patients, because GPs must be trained in the intervention (InPAct program). We therefore used cluster randomisation of GPs belonging to the same office or network of knowledge or care in order to avoid contamination bias. Second, it was difficult to use a parallel design as in a classic CRCT. Indeed, for logistical reasons, and because the workload and availability of GPs can be adversely affected at certain times of the year (due to winter epidemics, seasonal allergies, or high demand for back-to-school medical certificates in September), it was difficult to implement the program to all GPs simultaneously in the InPAct study. One advantage of the stepped wedge design is that the program will be introduced to GPs sequentially in the order assigned by randomisation. So, only some GPs must be trained simultaneously in the presentation of the booklet, over a particular time period. Third, since we obtained no arguments in favour of an adverse effect of the InPAct program during the pilot study [18], and since all GPs will eventually receive the intervention (which is a characteristic of the stepped wedge design versus a classic CRCT design), GPs are motivated to continue their participation until study’s end.

There are also statistical advantages to choosing a CRCT stepped wedge trial. First of all, GPs are their own controls in both control and intervention groups. This improves comparability in both groups. The CRCT stepped wedge design may also require fewer clusters than a CRCT parallel group design to ensure similar group comparability, in terms of population characteristics. This design therefore increases statistical power compared to parallel group design, because the program effect is estimated not only by between-cluster comparisons but also by within-cluster comparisons. This suggests that the use of a stepped wedge design should decrease patient sample size, compared to a classic CRCT parallel group design [17,20,32]. For the InPAct study, the number of patients to be included (1,200) was calculated to be strongly reduced with a stepped wedge design versus a classic CRCT design, in order to maintain the statistical power of 80% and a cluster number of eight. Additionally, the effects of time can be included in our choice of statistical model, which allows temporal changes in the effectiveness of the program to be considered [16,17,20]. Indeed, the opportunities arising from modelling the effects of time can be illustrated by considering the stepped wedge design as a multiple-arm parallel design, in which the research aims not only to assess intervention effects, but also to determine whether time of intervention affects the effectiveness of the intervention. Such time effects can include seasonal variations both in GP practice and in the reporting, occurrence and progression of ADEs. In general practice, the seasonal variability in the workload of GPs may negatively affect both their availability for providing health information and the effectiveness of the intervention for the patient. The behaviour of both GPs and patients could also be influenced by external factors that are independent of the study, such as a public campaign about a reporting system for ADEs. For the patients, diuretic antihypertensive-associated ADEs such as dehydration may be more serious in summer due to high temperatures. Even if including time as a random effect could be an advantage of the stepped wedge design, Hussey and Hughes have suggested that a relatively complex model might be required to take into account the time effect [20].

Finally, the InPAct study will provide additional knowledge about the effects of both an interactive program for GPs and patients, and an ADE reporting system for patients. If the InPAct program is found to be effective in increasing ADE reporting by hypertensive patients and in implementing corrective and preventive actions for improving patient safety in general practice, then this program could be implemented in other regions. Indeed, another advantage of the stepped wedge design is its faculty to test the feasibility of implementing an intervention in a certain number of regions prior to its generalisation. Moreover, other similar programs, using the same levers, could be implemented for other commonly prescribed drugs such as anticoagulants, psychotropics, analgesics, or antibiotics in primary care.

## Abbreviations

ADE: Adverse drug event; GP: General practitioner; InPAct: Information for Participating Actively; ANSM: French National Agency for the Safety of Medicines and Health Products; CRCT: Cluster randomised controlled trial; U: Unit of randomisation; e-CRF: Electronic case report form; Cv: Coefficient of variation.

## Competing interests

The authors declare that they have no competing interests.

## Authors’ contributions

The chief investigator of the study, MKG, as well as KP, AD and CC, were responsible for determining the research question, the study design, the methodology and the follow-up of the study; obtaining ethics approval; acquiring financial support, and writing the paper. MR contributed to the fine-tuning of the methodology and statistical analysis. SF, JPD and JB contributed to the follow-up of the study and to the recruiting of GP investigators. TV’s role is to carry out the pharmacovigilance evaluation. All authors helped draft and revise the manuscript and approved the final version.

## References

[B1] Kingston-Riechers J Ospina M Jonsson E Childs P McLeod L Maxted J Patient safety in primary care 2010 Edmonton, AB: Canadian Patient Safety Institute and British Columbia Patient Safety and Quality Council

[B2] Gandhi TK Weingart SN Borus J Seger AC Peterson J Burdick E Seger DL Shu K Federico F Leape LL Bates DW Adverse drug events in ambulatory care N Engl J Med 2003 348 1556 1564 10.1056/NEJMsa020703 12700376

[B3] Gurwitz JH Field TS Harrold LR Rothschild J Debellis K Seger AC Cadoret C Fish LS Garber L Kelleher M Bates DW Incidence and preventability of adverse drug events among older persons in the ambulatory setting JAMA 2003 289 1107 1116 10.1001/jama.289.9.1107 12622580

[B4] Smith PC Araya-Guerra R Bublitz C Parnes B Dickinson LM Van Vorst R Westfall JM Pace WD Missing clinical information during primary care visits JAMA 2005 293 565 571 10.1001/jama.293.5.565 15687311

[B5] Kripalani S Jackson AT Schnipper JL Coleman EA Promoting effective transitions of care at hospital discharge: a review of key issues for hospitalists J Hosp Med 2007 2 314 323 10.1002/jhm.228 17935242

[B6] WHO “Nine patient safety solutions” to help reduce the toll of health care-related harm affecting millions of patients worldwide 2007 Washington/Geneva: WHO Press 2 may 2007

[B7] De Langen J Van Hunsel F Passier A De Jong-van den Berg L Van Grootheest K Adverse drug reaction reporting by patients in the Netherlands: three years of experience Drug Saf 2008 31 515 524 10.2165/00002018-200831060-00006 18484785

[B8] Friedman SM Provan D Moore S Hanneman K Errors, near misses and adverse events in the emergency department: what can patients tell us? CJEM 2008 10 421 427 18826729 10.1017/s1481803500010484

[B9] McLernon DJ Bond CM Hannaford PC Watson MC Lee AJ Hazell L Avery A Yellow Card Collaboration Adverse drug reaction reporting in the UK: a retrospective observational comparison of yellow card reports submitted by patients and healthcare professionals Drug Saf 2010 33 775 788 10.2165/11536510-000000000-00000 20701410

[B10] Inch J Watson MC Anakwe-Umeh S Patient versus healthcare professional spontaneous adverse drug reaction reporting: a systematic review Drug Saf 2012 35 807 818 22928729 10.1007/BF03261977

[B11] Fifth Joint Task Force of the European Society of Cardiology et al European Guidelines on cardiovascular disease prevention in clinical practice (version 2012): the Fifth Joint Task Force of the European Society of Cardiology and Other Societies on Cardiovascular Disease Prevention in Clinical Practice (constituted by representatives of nine societies and by invited experts) Eur J Prev Cardiol 2012 19 585 667 22763626 10.1177/2047487312450228

[B12] Runciman WB Roughead EE Semple SJ Adams RJ Adverse drug events and medication errors in Australia Int J Qual Health Care 2003 15 Suppl 1 i49 i59 14660523 10.1093/intqhc/mzg085

[B13] Gurwitz JH Field TS Judge J Rochon P Harrold LR Cadoret C Lee M White K LaPrino J Erramuspe-Mainard J DeFlorio M Gavendo L Auger J Bates DW The incidence of adverse drug events in two large academic long-term care facilities Am J Med 2005 118 251 258 10.1016/j.amjmed.2004.09.018 15745723

[B14] Carbonin P Pahor M Bernabei R Sgadari A Is age an independent risk factor of adverse drug reactions in hospitalized medical patients? J Am Geriatr Soc 1991 39 1093 1099 1753048 10.1111/j.1532-5415.1991.tb02875.x

[B15] Brown C Hofer T Johal A Thomson R Nicholl J Franklin BD Lilford RJ An epistemology of patient safety research: a framework for study design and interpretation. Part 4. One size does not fit all Qual Saf Health Care 2008 17 178 181 10.1136/qshc.2007.023663 18519623

[B16] Brown CA Lilford RJ The stepped wedge trial design: a systematic review BMC Med Res Methodol 2006 6 54 10.1186/1471-2288-6-54 17092344 PMC1636652

[B17] Mdege ND Man M-S Taylor Nee Brown CA Torgerson DJ Systematic review of stepped wedge cluster randomized trials shows that design is particularly used to evaluate interventions during routine implementation J Clin Epidemiol 2011 64 936 948 10.1016/j.jclinepi.2010.12.003 21411284

[B18] Keriel-Gascou M Badet-Phan A Le Pogam M-A Figon S Letrilliart L Gueyffier F Buchet-Poyau K Duclos A Colin C Information and active participation of the patient with an interactive booklet in primary care prescription of antihypertensives Sante Publique 2013 25 193 201 23964544

[B19] Fink A Kosecoff J Chassin M Brook RH Consensus methods: characteristics and guidelines for use Am J Publ Health 1984 74 979 983 10.2105/AJPH.74.9.979 PMC1651783 6380323

[B20] Hussey MA Hughes JP Design and analysis of stepped wedge cluster randomized trials Contemp Clin Trials 2007 28 182 191 10.1016/j.cct.2006.05.007 16829207

[B21] Campbell MK Piaggio G Elbourne DR Altman DG Consort 2010 statement: extension to cluster randomised trials BMJ 2012 345 e5661 10.1136/bmj.e5661 22951546

[B22] Austin PC Estimating multilevel logistic regression models when the number of clusters is low: a comparison of different statistical software procedures Int J Biostat 2010 6 16 10.2202/1557-4679.1195 PMC2949382 20949128

[B23] Craig P Dieppe P Macintyre S Michie S Nazareth I Petticrew M Developing and evaluating complex interventions: the new Medical Research Council guidance BMJ 2008 337 a1655 10.1136/bmj.a1655 18824488 PMC2769032

[B24] Weingart SN Hamrick HE Tutkus S Carbo A Sands DZ Tess A Davis RB Bates DW Phillips RS Medication safety messages for patients via the web portal: the MedCheck intervention Int J Med Inform 2008 77 161 168 10.1016/j.ijmedinf.2007.04.007 17581772

[B25] Roughead E Pratt N Peck R Gilbert A Improving medication safety: influence of a patient-specific prescriber feedback program on rate of medication reviews performed by Australian general medical practitioners Pharmacoepidemiol Drug Saf 2007 16 797 803 10.1002/pds.1393 17476702

[B26] Fick DM Maclean JR Rodriguez NA Short L Heuvel RV Waller JL Rogers RL A randomized study to decrease the use of potentially inappropriate medications among community-dwelling older adults in a southeastern managed care organization Am J Manag Care 2004 10 761 768 15623266

[B27] Little P Dorward M Warner G Moore M Stephens K Senior J Kendrick T Randomised controlled trial of effect of leaflets to empower patients in consultations in primary care BMJ 2004 328 441 10.1136/bmj.37999.716157.44 14966078 PMC344265

[B28] Little P Somerville J Williamson I Warner G Moore M Wiles R George S Smith A Peveler R Randomised controlled trial of self management leaflets and booklets for minor illness provided by post BMJ 2001 322 1214 1216 1217 10.1136/bmj.322.7296.1214 11358775 PMC31621

[B29] Little P Griffin S Kelly J Dickson N Sadler C Effect of educational leaflets and questions on knowledge of contraception in women taking the combined contraceptive pill: randomised controlled trial BMJ 1998 316 1948 1952 10.1136/bmj.316.7149.1948 9641933 PMC28594

[B30] Dawes MG Kaczorowski J Swanson G Hickey J Karwalajtys T The effect of a patient education booklet and BP “tracker” on knowledge about hypertension. A randomized controlled trial Fam Pract 2010 27 472 478 10.1093/fampra/cmq048 20631056

[B31] Pernod G Labarère J Yver J Satger B Allenet B Berremili T Fontaine M Franco G Bosson JL EDUC’AVK: reduction of oral anticoagulant-related adverse events after patient education: a prospective multicenter open randomized study J Gen Intern Med 2008 23 1441 1446 10.1007/s11606-008-0690-1 18566863 PMC2518037

[B32] Pearson D Torgerson D McDougall C Bowles R Parable of two agencies, one of which randomizes ANNALS Am Acad Polit Soc Sci 2010 628 11 29 10.1177/0002716209351500

